# Cardiac thromboxane A2 receptor activation does not directly induce cardiomyocyte hypertrophy but does cause cell death that is prevented with gentamicin and 2-APB

**DOI:** 10.1186/2050-6511-15-73

**Published:** 2014-12-17

**Authors:** Chad D Touchberry, Neerupma Silswal, Vladimir Tchikrizov, Christopher J Elmore, Shubra Srinivas, Adil S Akthar, Hannah K Swan, Lori A Wetmore, Michael J Wacker

**Affiliations:** Muscle Biology Group, School of Medicine, University of Missouri-Kansas City, 2464 Charlotte Street, Kansas City, MO 64108 USA; Biochemistry/Molecular Biology Lab, Department of Health and Sport Sciences, University of Memphis, Memphis, TN 38152 USA; Department of Chemistry, William Jewell College, Liberty, MO 64068 USA

**Keywords:** U46619, TXA2, Cardiomyocytes, TP receptor, Cardiovascular disease, IP3

## Abstract

**Background:**

We have previously shown that the thromboxane (TXA2) receptor agonist, U46619, can directly induce ventricular arrhythmias that were associated with increases in intracellular calcium in cardiomyocytes. Since TXA2 is an inflammatory mediator and induces direct calcium changes in cardiomyocytes, we hypothesized that TXA2 released during ischemia or inflammation could also cause cardiac remodeling.

**Methods:**

U46619 (0.1-10 μM) was applied to isolated adult mouse ventricular primary cardiomyocytes, mouse ventricular cardiac muscle strips, and cultured HL-1 cardiomyocytes and markers of hypertrophy and cell death were measured.

**Results:**

We found that TXA2 receptors were expressed in ventricular cardiomyocytes and were functional via calcium imaging. U46619 treatment for 24 h did not increase expression of pathological hypertrophy genes (atrial natriuretic peptide, β-myosin heavy chain, skeletal muscle α-actin) and it did not increase protein synthesis. There was also no increase in cardiomyocyte size after 48 h treatment with U46619 as measured by flow cytometry. However, U46619 (0.1-10 μM) caused a concentration-dependent increase in cardiomyocyte death (trypan blue, MTT assays, visual cell counts and TUNEL stain) after 24 h. Treatment of cells with the TXA2 receptor antagonist SQ29548 and inhibitors of the IP3 pathway, gentamicin and 2-APB, eliminated the increase in cell death induced by U46619.

**Conclusions:**

Our data suggests that TXA2 does not induce cardiac hypertrophy, but does induce cell death that is mediated in part by IP3 signaling pathways. These findings may provide important therapeutic targets for inflammatory-induced cardiac apoptosis that can lead to heart failure.

## Background

Thromboxane A2 (TXA2) is a member of the prostaglandin family and is produced from prostaglandin H2 via thromboxane-A synthase activity. It has long been recognized that TXA2 levels are elevated in the circulation as a result of obesity [1], systemic inflammation [2] and myocardial ischemia [3–5]. In addition, TXA2 has been strongly implicated in mediating cardiovascular events, principally because of its well-characterized actions in inducing platelet aggregation and vasoconstriction [6, 7]. Because of these actions, antiplatelet agents such as aspirin have been used as a preventative therapy to reduce the risk of cardiovascular events [8]. While there is little doubt that TXA2 can play an *indirect* role in contributing to heart disease via vasoconstriction and platelet aggregation, the goal of our laboratory is to characterize the *direct* actions of TXA2 on the heart.

Previously, while investigating the ability of the TXA2 mimetic (U46619) to stimulate peripheral sensory neurons involved in autonomic nervous system reflexes in the anesthetized rabbit [9], we noted that left atrial injections of U46619 induced ventricular arrhythmias. These arrhythmias were independent of changes in coronary blood flow, systemic vasoconstriction, and without the induction of myocardial ischemia [10], which indicated that the effect was a direct action on the heart by U46619. To further elucidate the mechanisms responsible for these arrhythmias, we found that rabbit ventricular cardiomyocytes expressed TXA2 receptors (TXA2Rs) and antagonism of TXA2R eliminated the arrhythmias [10]. It is well known in platelets and smooth muscle cells that stimulation of TXA2R activates phospholipase C (PLC), increases inositol trisphosphate (IP3) production, and releases Ca^2+^ from intracellular stores [11–15]. Our laboratory and others have also found that U46619 stimulation of TXA2Rs on adult ventricular cardiomyocytes (AVCMs) increases intracellular Ca^2+^ [16–19]. Crucially, our laboratory found that pre-treatment with an inhibitor of IP3 formation, gentamicin, or an inhibitor of IP3 receptors, 2 aminoethyl diphenylborate (2-APB), not only prevented the increase in intracellular Ca^2+^*in vitro*, but also inhibited the formation of U46619 induced arrhythmias *in vivo* [10, 16]. Because intracellular Ca^2+^ homeostasis is critical to normal heart function and disruption of intracellular Ca^2+^ not only triggers arrhythmias [20], but also cardiac hypertrophy [21, 22] and cell death [23], we wanted to investigate other potential roles TXA2 may play in the myocardium.

Previous research in rodents has demonstrated an important role for TXA2 signaling as being associated with reduced ejection fraction [24–26]. However, it is unclear if the reduced cardiac function is due to TXA2 inducing pathological hypertrophy, cardiomyocyte cell death, or a combination of these leading to remodeling. Various reports have provided evidence supporting the potential for both possibilities [24, 26–28]. Specifically, Zhang *et al*. [26] have shown that overexpression of the GTP binding protein, Gh, induces cyclooxygenase 2 (COX2), TXA2 synthase, and TXA2R expression and an increase in TXB2 (the metabolite of TXA2). They observed an increase in left ventricular mass and heart weight to body weight ratio, but also showed an increase in fibrosis and apoptosis. These authors concluded that TXA2 may augment cardiac hypertrophy, but could also play a role in cell death during remodeling. In a study by Lin *et al*. [24], iron-overloaded mice (which have increased cardiac TXA synthase and cardiac TXB2 levels) had increased heart weight to body weight ratio in wild type, but not TXA2 synthase null mice. While they did not see ventricular wall hypertrophy, they did observe cardiac fibrosis which was reduced in the TXA2 synthase null mice. These results are complicated by all of the factors that go along with *in vivo* studies. Specifically, it is possible that other factors that were elevated in these studies, PGF_2α_, PGI_2_, and TNF_α_, via COX2 activation or downstream of TXA2R activation may have contributed to the cardiac phenotype [24, 26]. Nevertheless, the results indicate that TXA2 plays a role in cardiac function/remodeling.

What remains to be clarified is what type of response TXA2 is most likely to directly induce on cardiomyocytes in absence of other factors. Therefore, we wanted to analyze the effects of cardiac TXA2R stimulation on both hypertrophy and cell death in the same controlled study utilizing the same agonist and concentrations to determine which action is favored. Additionally, no studies have looked at IP3 inhibition to target the direct cardiac TXA2 effects beyond our study with arrhythmias. It is possible that this same signaling pathway is involved in multiple cardiac effects mediated by TXA2R stimulation. Since TXA2 has been shown to be associated with reduced ejection fraction, it is necessary that we identify the role of TXA2 in cardiac remodeling and identify potential therapeutic targets for inhibition. Therefore, we sought to determine if *in vitro* treatment of cardiac tissue and isolated cells with a TXA2 mimetic alone would: 1) induce cardiac hypertrophy and/or cell death, and 2) determine if deleterious changes could be attenuated with gentamicin or 2-APB treatment, as we have previously shown with U46619 induced arrhythmogenesis.

## Methods

### Materials

U46619, SQ29548, and 2-APB were purchased from Cayman Chemical (Ann Arbor, MI). Hanks balanced salt solution (HBSS) and Fura-2 AM were obtained from Invitrogen (Carlsbad, CA). Enzymes for cardiomyocyte digestion were purchased from Worthington Biochemical (Lakewood, NJ). Total RNA Isolation kits were purchased from IBI Scientific (Peosta, IA), and the real-time reverse-transcriptase polymerase chain reaction (RT-PCR) was performed using a TaqMan RNA-to-CT 1 step kit and primers and probes from ABI (Carlsbad, CA). Primary antibody for TXA2R was purchased from Abcam (Cambridge, MA). Gentamicin and fetal bovine serum was obtained from Sigma-Aldrich (St. Louis, MO). DeadEnd Fluorometeric TUNEL stain was purchased from Promega (Madison, WI). All remaining reagents were purchased from Fisher Scientific.

### Animals

Twelve-week-old male CD-1 mice (Harlan Laboratories, Madison, WI) were used in experiments using exogenous U46619. All mice were housed in a temperature-controlled (22 ± 2°C) room with a 12:12 h light-dark cycle. Animals were fed *ad libitum*. All protocols were approved by the Animal Care and Use Committee of the University of Missouri-Kansas City.

### Isolation and culture of primary mouse AVCMs

Following cervical dislocation of mice, the heart was rapidly excised, extraneous tissue was removed, and the aorta was cannulated under a dissection microscope. AVCMs were isolated, utilizing retrograde perfusion via a proprietary procedure developed in our laboratory with Worthington Biochemical (Lakewood, NJ) as previously described [29]. Briefly, hearts were retrograde perfused through the aorta using a Langendorff perfusion apparatus with Ca^2+^-free perfusion buffer (3 ml/min) for 4 min and then switched to a digestion buffer containing collagenase II (18,000 U), papain (20 U), DNase (2,000 U) and 2,3-butanedione monoxime (BDM; 10 mM) for 8-10 min at 37°C. The heart was removed from perfusion, cut into pieces and pipetted gently to disperse cells in suspension. Calcium-tolerant myocytes were then plated onto chamber slides previously coated with 10μg/ml laminin and kept in L15 medium (10% FBS, 2 mM L-glutamine) with BDM (10 mM) for 1 h for attachment. After 1 h, serum-containing media was switched to serum-free culture media (L15, 0.2% BSA, 2 mM L-glutamine, 10 mM BDM) for the remainder of the experiments.

### HL-1 cardiomyocytes and flow cytometry

HL-1 cardiomyocytes were utilized since they maintain phenotypic characteristics of adult myocytes [30], and have been used previously in models of cardiac hypertrophy using flow cytometry [29, 31–34] and cell death [35–38]. HL-1 cardiomyocytes were plated (5,000/cm^2^) in flasks precoated with 0.00125% fibronectin and 0.02% gelatin. Cells were cultured for 24 h in Claycomb media (supplemented with 10% FBS, 2 mM L-glutamine, 0.1 mM norepinephrine, 0.3 mM ascorbic acid, 100 U/ml penicillin, and 100 mg/ml streptomycin). Cells were treated with vehicle (methyl acetate) or U46619 (0.1, 1, 5 and 10 μM) for 48 h in a minimal media (0.5% FBS, 2 mM L-glutamine, and without penicillin-streptomycin, and norepinephrine) prior to analysis. Cells were collected and analyzed for changes in cells size by flow cytometry using FACSCalibur forward scatter (FSC-H). FSC-H analysis of > 10,000 live gated cells/sample from independent experiments were normalized to vehicle controls and averaged.

### Gene expression

Total RNA was isolated from serum starved (24 h) HL-1 cardiomyocytes and AVCMs and real-time RT-PCR was performed using the Corbett Rotor-Gene 6000 (Qiagen, Valencia, CA). Gene expression was conducted using 2^-ΔΔCT^ analysis against β-actin. β-actin was chosen as a housekeeping gene since U46619 treatment had minimal changes in gene expression compared with vehicle-treatment. GAPDH comparisons also yielded similar results as that with β-actin.

### Total protein & western blot

Cardiac muscle strips and AVCMs were lysed in ice-cold cell extraction buffer (Invitrogen), as described previously [29, 39]. Total protein concentration of the samples was determined by use of the micro bicinchoninic acid protein assay (Fisher Scientific). Protein from cardiac muscle strips were normalized to tissue weight (μg/mg). Protein extracts from AVCMs (20-50 μg) were run on a 10% SDS-PAGE gels and proteins were transferred to PVDF membranes using standard techniques to visualize TXA2R protein. The membranes were blocked in 5% BSA for 1 h at room temperature; primary antibodies were diluted 1:1000 in 5% BSA and incubated overnight. Blots were incubated in HRP-conjugated secondary antibody at 1:20,000 in 5% milk for 1 h at room temperature. Bands were visualized by enhanced chemiluminescence.

### Ca^2+^ imaging

Serum starved HL-1 cells and isolated AVCMs were loaded at room temperature with the Ca^2+^ indicator dye, fura-2 AM (Invitrogen; 2μM) in 0.025% pluronic F-127, for 20 min. Cells were washed 3 times in HBSS and allowed to de-esterify for 10 min at room temperature. Intracellular Ca^2+^ levels were measured with an inverted microscope with fluorescent imaging capabilities [Olympus IX51 (Olympus, Melville, NY) and Hamamatsu Orca-ERGA charge-coupled device cameras (Hamamatsu, Bridgewater, NJ), Semrock Bright Line filter set (Semrock, Rochester, NY), EXFO X-cite metal halide light source (EXFO, Mississauga, ON, Canada), and Slidebook ratiometric software (Intelligent Imaging Innovations)]. U46619 was perfused into dishes and the ratiometric responses were recorded. Data was only included if a positive control KCl response was greater than a 100% increase above the baseline fluorescence.

### Tissue culture

CD-1 mouse ventricular tissue strips were isolated from mouse hearts and used for the tissue culture experiments. Hearts were quickly excised and placed into an ice-cold cardioprotective medium that included the addition of BDM (30 mM), as we have described previously [29, 40]. Tissue cultures were treated with vehicle, or increasing dosages of U46619 (0.1, 1, and 10 μM) or the positive control, FGF23 (35 pM), and then analyzed for changes in total protein content using the micro bicinchoninic acid protein assay as described above.

### Cell death assays

HL-1 cardiomyocytes were plated in full Claycomb medium as previously described. Twenty-four hours following plating, cells were switched to a serum and norepinephrine free Claycomb medium. 24 h following serum starvation, myocytes were treated with U46619 (0.01, 0.1, 1, 5, 10 μM) for 24 h in the serum and norepinephrine free condition.

Lactate dehydrogenase (LDH) assay (Roche, Indianapolis, IN): HL-1 cells were plated in a 96-well plate and treated for 4-6 h with U46619 (1 and 10 M). Optical density was measured at 490 and 690 nM wavelengths and the difference was calculated to determine LDH in the incubation media. Minimum or baseline LDH activity was determined using a plate of cells without U46619 treatment. Maximum LDH activity was determined using untreated cells that were permeabilized with 2% Triton-X. The percent of total LDH release was then calculated for each treatment (experimental value- minimum)/(maximum-minimum)*100.

Trypan blue assay: Following U46619 treatment of HL-1 cardiomyocytes, medium was removed and saved, and the attached cells trypsinized (0.05%) for 3 min at 37°C. Medium and trypsinized cells were placed into a tube with soybean trypsin inhibitor and pelleted at 500 g for 3 minutes. The cell pellet was resuspended in 1 ml of HBSS. 30 μL of cell suspension was added to 270 μl of 0.4% trypan blue for 5 min. 10 μL of trypan blue stained cells was added to the hemocytometer and trypan positive and negative cells were counted by an investigator blinded to the treatment conditions. This process was repeated in triplicate for each condition and the experiment was replicated on 4 separate occasions.

MTT Assay (3-[4,5-dimethylthiazol-2-yl]-2,5-diphenyltetrazolium bromide): HL-1 cardiomyocytes were cultured in a 96 well plate. After U46619 treatment, survival of myocytes was determined as previously described [41]. Briefly, medium was removed from each well and replaced with 90 μL of fresh phenol-red free medium. 10 μL of 12 mM MTT stock solution (dissolved in saline) was added to each well. Cells were incubated for an additional 3 h in the presence of MTT (0.5%). After incubation 75 μL was removed from the wells and the reaction was terminated via the addition of 50 μL of DMSO on a swirling rocker. Cells were then incubated for 10 min at 37°C and the absorbance was read at 540 nm in a spectrophotometer. The amount of blue formazan dye generated from MTT is proportional to the number of live cells, thus the assay is a viability test. Values of the reaction were obtained after subtraction of matched blanks and the optical densities (ODs) of the vehicles were taken as 100% for comparisons with values for U46619 treated samples. Percent of dead cells was calculated with the following equation (100-((OD_U46619_/OD_veh_)*100)).

Primary cardiomyocytes: Equal numbers of isolated AVCMs were plated and allowed to attach to the laminin substrate for 1 h as previously described. After 1 h, the cells were washed three times in L15 containing BDM (10 mM) to rinse away unattached cells, debris and serum. Next, the serum-containing media was switched to serum-free culture media (L15, 0.2% BSA, 2 mM L-glutamine, 10 mM BDM) for 4 h. Then the cells were treated with increasing concentrations of vehicle or U46619 (1, 5 and 10 μM) for 24 h.

Cell counts of primary AVCMs per field: Immediately following the addition of vehicle and U46619, we took a cell count (0 h) of AVCMs. Rod-shaped morphology, with clear striations were the criteria used for identifying viable cells, whereas round-shaped myocytes, loss of striation or membrane blebbing were considered non-viable cells. After 24 h incubation, the cells were washed three times in L15 medium containing BDM (10 mM) to rinse away dead cells and debris and recounted (24 h). Using the 10x objective on an inverted microscope, five fields were counted per treatment condition and the number of rod-shaped, striated myocytes was averaged in 3 independent experiments by an investigator blind to the conditions. We then calculated the ratio of healthy cells in the U46619 wells/vehicle wells at 0 h and 24 h. Percent change was determined by the formula ((24 h ratio – 0 h ratio/ 0 h ratio)*100).

TUNEL Stain: isolated AVCMs were plated on laminin coated plastic chamber slides and treated as previously described. DeadEnd Fluorometric TUNEL stain was used to label nuclear DNA fragmentation and was performed according to manufactures recommendations. Briefly, AVCMs were fixed in 10% formaldehyde in PBS for 25 min. Slides were rinsed in PBS and then permeabilized in 0.2% Triton X-100 in PBS for 5 min. Slides were rinsed in PBS and then exposed to equilibration buffer for 10 min. Cells were then labeled with TdT reaction mix and covered with plastic coverslips and incubated for 60 min at 37°C. Plastic cover slips were removed and slides were immersed in 2x SSC buffer for 15 min. After washing slides in PBS, slides were covered in mounting medium with DAPI stain (Vectashield). TUNEL positive cells were identified using an inverted microscope with fluorescent imaging capabilities. Three to five areas at random were selected and TUNEL positive and negative cells were counted.

### Statistical analysis

All statistical procedures and graphs were performed with GraphPad Prism 5 (La Jolla, CA). Data are presented as means ± SEM. Data were compared using either a paired *t*-test or a one-way analysis of variance, and significance was set at the *p* < 0.05 level. When necessary, the one-way analysis of variance was followed with appropriate post hoc tests. A Bonferroni post hoc adjustment was used to correct for two to three comparisons to avoid type I error. In cases where we made more than three comparisons, we utilized a Tukey post hoc adjustment to avoid type II error. FSC data was analyzed using FlowJo version 8.8.6 probability binning population comparison software (Tree Star) using a modified Cox Chi Squared Test [T(X)]. Value of T(X) > 4 implies that the two distributions are different with a *p* < 0.01.

## Results

### Cardiac TXA2 receptor expression and activation

**Figure 1:**
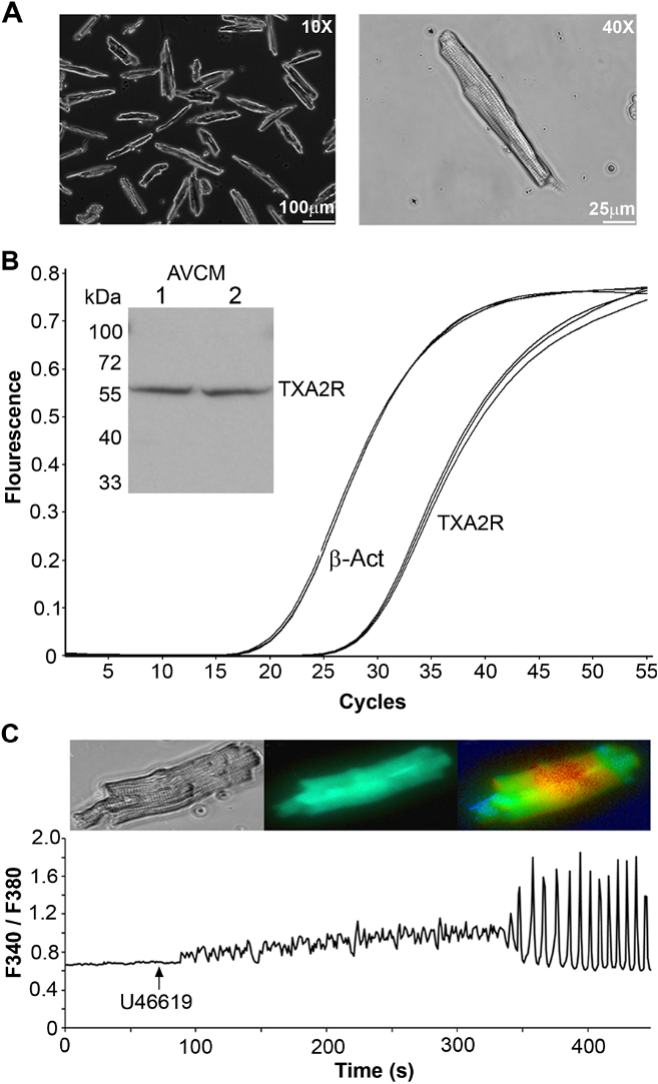
**TXA2R mRNA and protein are present in AVCMs. A**. 10x and 40x images of isolated AVCMs from male mice after 24 h in culture. Cells remain in high density; maintain membrane integrity and show clear striations associated with healthy cardiomyocytes following 24 h in culture. **B**. Real-time RT-PCR of RNA isolated from AVCMs showing the presence of TXA2R. Similar TXA2R gene expression was also observed with HL-1 cells. Inset shows TXA2R protein from isolated AVCMs detected by western blot. **C**. Representative data of 10 μM U46619-induced increases in intracellular Ca^2+^ in cardiomyocytes as measured by Fura-2 AM. This demonstrates that the TXA2R in mouse AVCMs is functional and behaves similarly to our previous reports in the rabbit [16].

Figure 1A shows mouse primary AVCMs following isolation and plating. Our cardiomyocyte isolation results in a high yield of long, striated and quiescent cardiomyocytes that maintain their morphology up to 48 h in culture. Figure 1B demonstrates positive detection of TXA2R mRNA and protein from isolated AVCMs (n = 2-3). We have also observed expression of TXA2R mRNA in HL-1 cells. There was a 3.2 fold higher expression of TXA2R in primary cardiomyocytes compared to HL-1 cardiomyocytes using β-actin as the reference gene and 2.7 fold higher expression using GAPDH as the reference gene. To determine if the TXA2R was functional, we conducted calcium imaging on mouse primary AVCMs. We have previously demonstrated that U46619 increases intracellular Ca^2+^ in rabbit cardiomyocytes [16] and have found that the cardiomyocytes from mice behave similarly to U46619 treatment as to what we have previously reported in the rabbit. As expected, the representative AVCM shown in Figure 1C displayed an increase in intracellular Ca^2+^ and had large Ca^2+^ oscillations following perfusion with U46619 (10 μM). U46619 also increased calcium levels in HL-1 cells (from 0.51 ± 0.2 at baseline to 1.01 ± 0.16 at the peak increase; n = 14 cells, *p* < 0.05).

### Markers of cardiac hypertrophy with exogenous U46619

**Figure 2:**
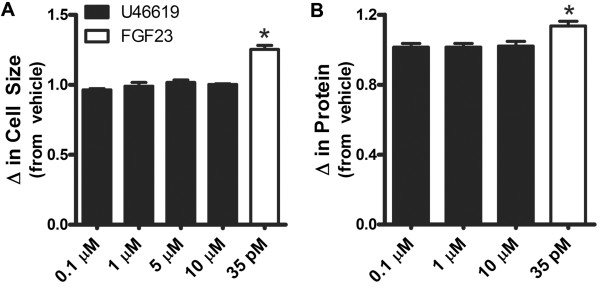
**U46619 does not induce hypertrophy or increase protein synthesis. A**. Flow cytometry forward-scatter (FSC-H) data was performed on more than 10,000 live gated cells/sample (n = 3). HL-1 cardiomyocytes were treated for 48 h with vehicle, increasing concentrations of U46619 (0.1-10 μM) [T(X) <4; *p* > 0.01], or a positive control, FGF23 (35 pM) [T(X) = 61, p < 0.01]. Results from independent experiments were normalized to vehicle controls and averaged and show that U46619 did not increase cell size (*p* > 0.05) while FGF23 increased cell size compared to vehicle (*p* < 0.05). **B**. Total protein concentration of ventricular muscle strips did not increase following 48 h treatment with U46619 (0.1-10 μM; n = 8; *p* > 0.05), but did increase after treatment with FGF23 (35 pM; n = 5; *p* < 0.05). *Statistical difference from vehicle.

**Figure 3:**
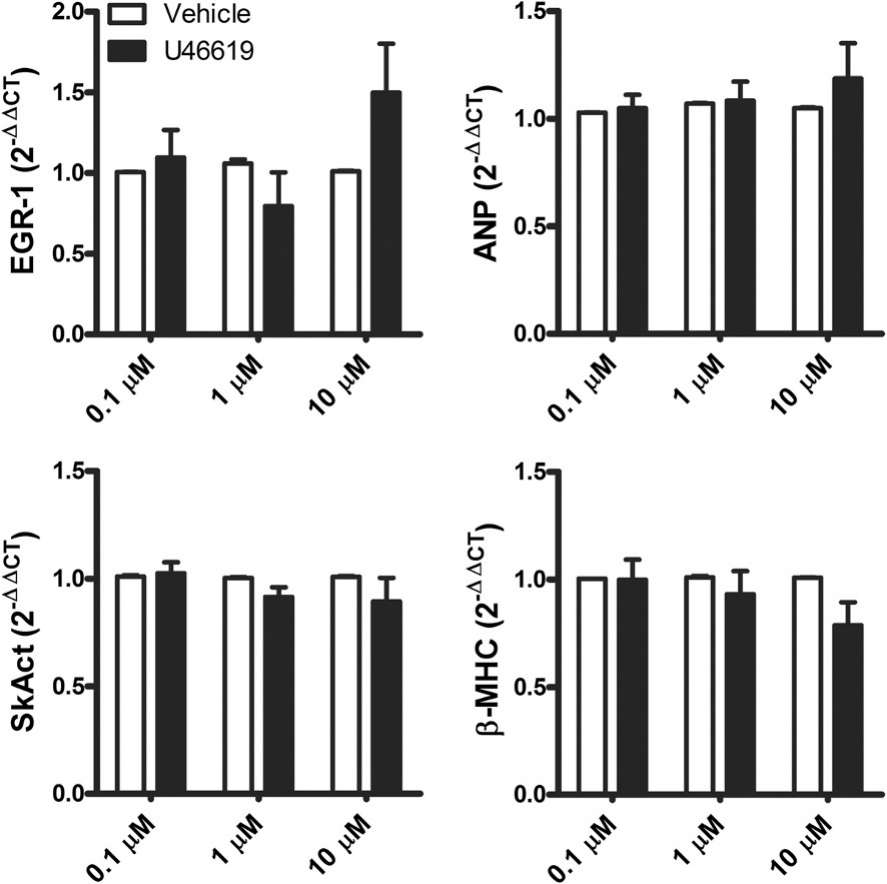
**U46619 does not increase gene markers associated with pathological hypertrophy.** Exposing AVCMs to increasing concentrations of U46619 (0.1-10 μM) did not increase the expression of early growth response 1 (EGR1) gene or the hypertrophy-associated genes atrial natriuretic peptide (ANP) after 24 h when compared to vehicle (*p* > 0.05). Forty-eight-hour treatment with U46619, did not increase the expression of other hypertrophy genes β-myosin heavy chain (β-MHC) and skeletal muscle α-actin (SkAct) when compared to vehicle (n = 4-5; *p* > 0.05). There was also no significant increase in these genes with U46619 treatment in HL-1 cardiomyocytes.

Since U46619 increases intracellular Ca^2+^ which can induce hypertrophy, we began this series of experiments by testing the hypothesis that U46619 treatment would induce cardiac hypertrophy. Increasing concentrations of U46619 (0.1, 1, 5, and 10 μM) for 48 h did not increase HL-1 cell size as measured via flow cytometry (forward scatter; FSC) [T(X) <4, *p* > 0.01; Figure 2A]. FGF23 was used as a positive control, as our lab has previously shown it to induce hypertrophy and increase forward scatter (FSC) in the HL-1 cell line [29]. FGF23 (35 pM) exposure for 48 h increased FSC by 25% [T(X) = 61, *p* < 0.01; Figure 2A]. In addition, U46619 treatment (0.1, 1, 10 μM) did not increase protein synthesis in ventricular muscle strips Figure 2B; *p* > 0.05; however, FGF23 (35 pM) exposure did result in a 14% increase in protein content (Figure 2B; *p* < 0.05), as we have previously shown [29]. Moreover, we determined the effect of U46619 on pathological gene markers of cardiac hypertrophy in AVCMs. Twenty-four h exposure to U46619 (0.1, 1, and 10 μM) did not result in statistically significant changes in the expression of early growth response 1 (EGR-1), atrial natriuretic peptide (ANP), skeletal muscle α-actin (SkAct) or β-myosin heavy chain (β-MHC) when compared to vehicle treated AVCMs (Figure 3; *p* > 0.05). We also did not observe significant changes in β-MHC (1.14 ± 0.07 fold), ANP (1.39 ± 0.31 fold), or SkAct (1.8 ± 0.75 fold) expression in HL-1 cells after 10 μM U46619 treatment compared to vehicle (P > 0.05; n = 4). This evidence does not support the hypothesis that U46619 can directly induce cardiac hypertrophy at these concentrations.

### Exogenous U46619 induces cell death

**Figure 4:**
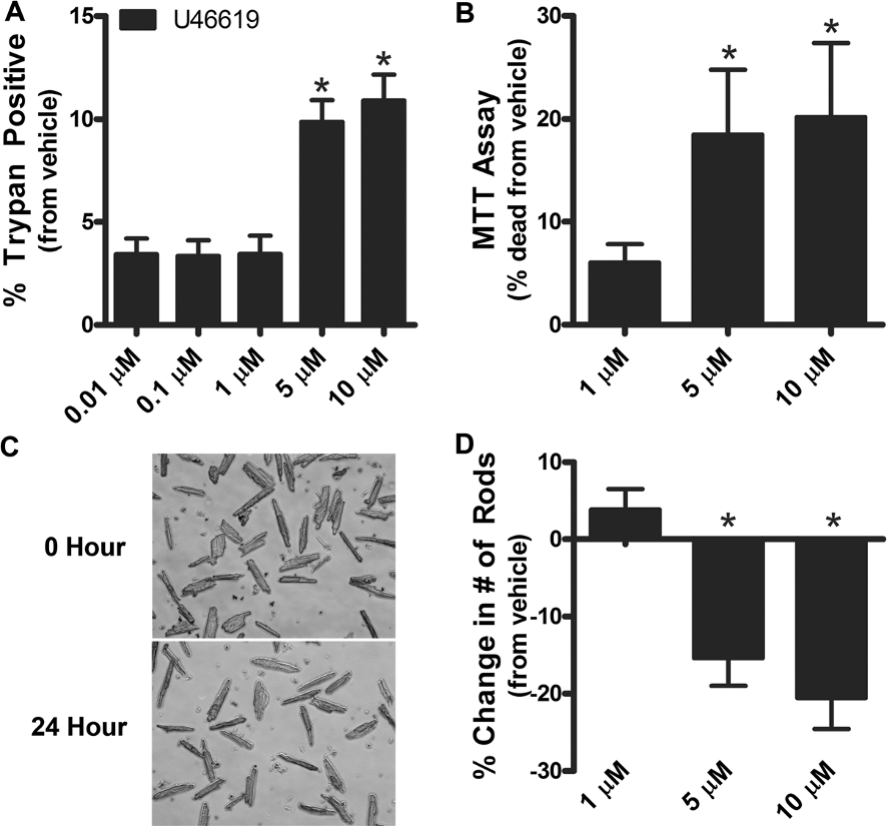
**U46619 increases cell death in cardiomyocytes. A**. HL-1 cardiomyocytes were incubated with increasing concentrations of U46619 (0.1-10 μM) and vehicle for 24 h. U46619 increased cell death at 5 and 10 M as measured by trypan blue staining (n = 3; *p* < 0.05). **B**. HL-1 cardiomyocytes were incubated with increasing concentrations of U46619 (1-10 μM) and vehicle for 24 h and viability was also determined by the MTT assay. A decrease in MTT staining was noted at 5 and 10 μM U46619 which indicates a decrease in metabolism and cell viability. Data were presented in terms of increased cell death from vehicle (n = 4, *p* < 0.05). **C**. Primary AVCMs were incubated with increasing concentrations of U46619 (1-10 μM) and vehicle for 24 h (image shows AVCMs treated with 10 μM U46619). Culture plates were washed and remaining cells counted (n = 3). Rod-shaped morphology, with clear striations were the criteria used for identifying viable cells, whereas round-shaped myocytes, loss of striation or membrane blebbing were considered non-viable cells. **D**. A reduction in the number of healthy rod shaped cardiomyocytes was seen at 5 and 10 μM U46619 (n = 3; *p* < 0.05). *Statistical difference from vehicle.

To determine if increasing concentrations of U46619 induced cell death, we analyzed various cell death assays. We did not observe an increase in LDH leakage at 4-6 hours of 1 and 10 μM U46619 in HL-1 cells (0.8 ± 0.8% and 0.4 ± 0.7% respectively of total LDH; n = 3) suggesting that there was minimal immediate necrosis or membrane damage. However, using trypan blue staining, we did observe a significant increase in trypan blue positive cells as compared to vehicle treatment at 5 and 10 μM concentrations of U46619 after 24 h (Figure 4A; *p* < 0.05). To verify the trypan blue staining, we analyzed an MTT assay of cell viability. Both 5 and 10 μM concentrations of U46619 induced a loss of metabolic activity at 24 h indicative of loss of cell viability (Figure 4B; *p* < 0.05). Further, we tested the effect of high doses of U46619 on cell death in primary AVCMs. Both 5 and 10 μM concentrations of U46619 resulted in a loss of healthy striated AVCMs at 24 h (Figure 4C; P < 0.05).

### TUNEL positive AVCMs with exogenous U46619

**Figure 5:**
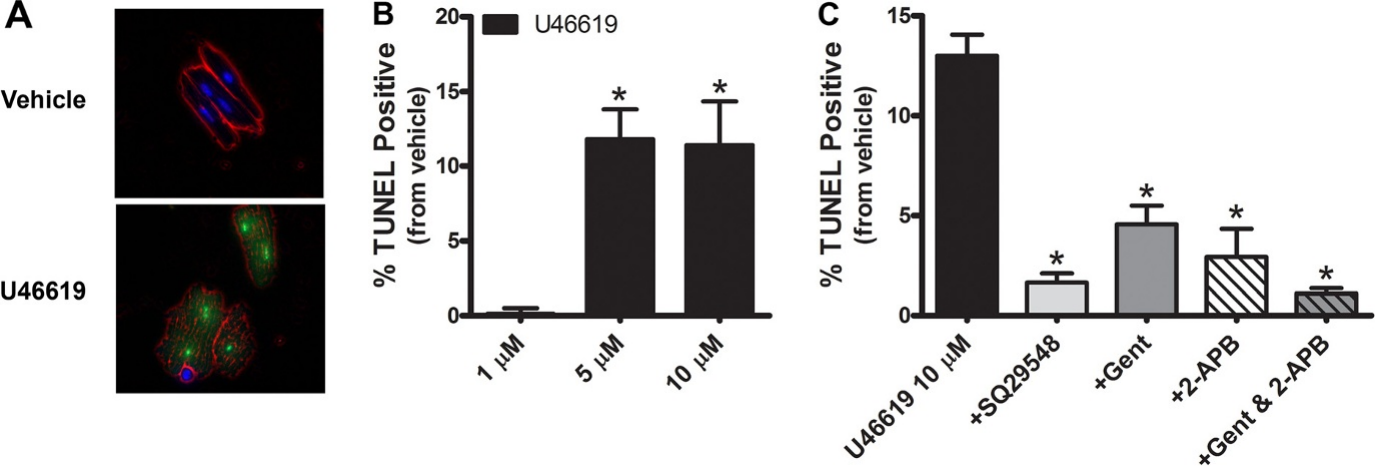
**U46619-induced DNA fragmentation is receptor mediated and inhibited with gentamicin and 2-APB. A**. Image of TUNEL positive AVCMs in culture. AVCMs were cultured in the absence of serum for 24 h during treatment with U46619 (0.1-10 μM) or vehicle and then stained with TUNEL (green nuclei) and DAPI (blue nuclei). Cell membranes were pseudo-colored to enhance visualization (red). **B**. Summary data showing increasing concentrations of U46619 (0.1-10 μM) increased the number TUNEL positive cardiomyocytes when compared to vehicle (n = 4; *p* < 0.05). *Statistical difference from vehicle. **C**. Summary data showing that pretreatment with a TXA2R antagonist (SQ29548; 10 μM), IP3 inhibitor (gentamicin, 10 μM), IP3R antagonist (2-APB, 10 μM), or combined IP3 signaling inhibitors (gentamicin + 2-APB) significantly reduced the number of TUNEL positive cardiomyocytes as a result of U46619 (10 μM) treatment (n = 4; *p* < 0.05). *Statistical difference from 10 μM U46619. Similar results to the TUNEL staining were also found with manual cell counts of viable AVCMs.

To determine if increasing concentrations of U46619 induced DNA fragmentation, we utilized TUNEL staining in AVCMs. All cells were stained with DAPI (blue) to identify nuclei and counterstained with fluorescein-12-dUTP (green) at 3’-OH DNA ends using the terminal deoxynucleotidyl transferase enzyme (Figure 5A). Exposure to U46619 (5 and 10 μM) increased the number of TUNEL positive AVCMs (Figure 5B; *p* < 0.05). We were able to significantly reduce U46619 induced DNA fragmentation by pre-treating AVCMs with SQ29548 (TXA2 receptor antagonist), gentamicin (inhibitor of IP3 formation), or 2-APB (inhibitor of IP3 receptors) (Figure 5C; *p* < 0.05). We also conducted manual cell counts of viable AVCMs and found similar results to the TUNEL assay. U46619 at 10 μM reduced viable cells by -20.3 ± 1.7% compared to vehicle, which was prevented by pretreatment with SQ29548 (-1.4 ± 3.0%*), gentamicin (-4.3 ± 6.2%*), and 2-APB (-1.2 ± 1.9%*) (n = 3; *P < 0.05 compared to U46619 treatment alone).

## Discussion

TXA2 is an important inflammatory mediator and could have significant direct actions on the heart during obesity and ischemia/reperfusion to potentially promote remodeling and heart failure. TXA2 levels are elevated during both myocardial ischemia and reperfusion [5, 42–44] with levels specifically increasing over 350% in the coronary sinus during myocardial ischemia [5]. Additionally, serum levels of TXA2 are known to increase in patients with obesity by 60% [1] and during heart failure by 235% [45]. There have been several basic science investigations that strongly suggest that TXA2 and TXA2R signaling are critically important in mediating the development of heart failure [24, 26, 46–48] and inhibition of TXA2 signaling has been shown to prevent cardiac remodeling [25] and improve cardiac function [49]. Nevertheless, how TXA2R activation may specifically promote remodeling of the heart has not been fully elucidated. Since cardiac TXA2R activation increases intracellular calcium it is possible that TXA2 may activate hypertrophic signaling or lead to cell death. Our current study was aimed to help elucidate and differentiate the direct actions of TXA2 on the myocardium in stimulating hypertrophy or cell death.

In order to investigate the direct effects of TXA2 on mouse hearts, we first verified the expression and function of TXA2Rs in AVCMs. Because the heart contains other cell types (smooth muscle cells and fibroblasts), we tested isolated AVCMs from mice. We found the presence of TXA2R mRNA and protein in AVCMs. These results are consistent with our findings in HL-1 cells, our previous observations of cardiac TXA2R expression from rabbit whole heart and single cell cardiomyocytes [16], and by others in cardiac tissue of other species [50, 51]. Exposure of the AVCMs or HL-1 cells to U46619-induced changes in intracellular Ca^2+^, which is also in agreement with our previous findings in adult rabbit cardiomyocytes [16], and by others in neonatal rat cardiomyocytes [18, 19]. In addition to increasing the intracellular Ca^2+^, U46619 increased the frequency of spontaneous Ca^2+^ oscillations. This correlates well with our previous work demonstrating that U46619 induces arrhythmias [16] and is supported by another study that demonstrated that U46619 was able to increase the beating rate and chaotic activity in neonatal rat cardiomyocytes [52]. These data provide additional support that mouse AVCMs express TXA2Rs and that these receptors are functional.

To test the hypothesis that stimulation of cardiac TXA2Rs can induce cardiac hypertrophy, we treated HL-1 cardiomyocytes with increasing concentrations of U46619. We found no change in cardiomyocyte cell size following exposure to U46619 as determined by flow cytometry. U46619 also did not alter protein synthesis, or the expression of genes associated with pathological hypertrophy, EGR-1, ANP, SkAct, or β-MHC. These results are somewhat surprising given that many other Gq-protein linked agents such as angiotensin II, norepinephrine, and endothelin-1 have long been known to induce cardiac hypertrophy [53, 54]. However, our findings are in agreement with a previous study utilizing rat neonatal cardiomyocytes that found that U46619 treatment (1 μM) only modestly increased the hypertrophy score (33%) and did not increase a hypertrophy marker, ANP [27]. This was in contrast to PGF_2α_ (1 μM) which dramatically increased the hypertrophy score (133%) and increased ANP expression. Our results are in contrast to Zhang *et al.* [26], with the overexpressed Gh mouse that displayed increased COX-2 and TXA2 levels. They observed an increase in left ventricular hypertrophy, which they attributed to cardiac TXA2R activation [26]. This group blocked the response with a TXA2R antagonist, but it remains possible that other prostaglandins such as PGF_2α_ or other paracrine factors may be involved in the hypertrophic response. It is also possible that TXA2 can activate both cell death and hypertrophy pathways and if no other factors are present, cell death predominates. If anti-apoptotic pathways are activated by other factors, then hypertrophy may be observed.

Analyzing U46619 treatment and cell death, our data in HL-1 cardiomyocytes showed an increase in trypan positive cells and reduced viability with an MTT assay confirming a significant increase in cell death at 24 h. Moreover, we supported these findings in AVCMs using visual cell counts of viable cells and also found that U46619 induced DNA fragmentation via TUNEL assay. These effects were TXA2R mediated as we were able to prevent the DNA fragmentation as well as overall cell death with the use of the TXA2R antagonist SQ29548. Collectively, these data convincingly show that TXA2 induces cell death.

We did not observe an increase in LDH leakage in cardiomyocytes up to 6 h, which is in agreement with a previous study that did not observe LDH leakage by U46619 treatment for up to 4 h in neonatal rat cardiomyocytes [18]. This would indicate that U46619 is likely not mediating immediate necrosis or oncosis. It is possible that the cell death observed at 24 h is mediated via apoptosis since TXA2R activation has been shown to induce apoptosis in endothelial cells [55], renal tubule cells [56], and immature thymocytes [57]. Shizukuda and Buttrick [28] have shown that the TXA2R agonist, IBOP, induced DNA fragmentation in adult rat cardiomyocytes at 24 h similar to our findings as well as decreased AKT activity and the authors concluded that TXA2R activation induces apoptosis. However, other mechanisms such as autophagy have not been analyzed in association with TXA2R activation. Therefore, additional studies will likely be needed to confirm the precise mechanisms responsible for TXA2-mediated cardiomyocyte death.

In an effort to eliminate the TXA2-induced cell death, we treated cells with gentamicin, 2-APB, or both drugs in combination. Gentamicin and 2-APB were selected for two reasons. First, TXA2R activates the Gq protein and can stimulate PLC, leading to IP3 formation, and subsequent increases in intracellular Ca^2+^ in cardiomyocytes [16–18]. Second, gentamicin and 2-APB have been widely used to inhibit the formation of IP3 or block IP3 receptors respectively. Gentamicin inhibits IP3 release by binding to and sequestering its precursor, phosphatidylinositol 4,5-bisphosphate [58], and has been used both *in vitro* and *ex vivo* with other known stimulators of the Gq/PLC pathway (i.e., thrombin, norepinephrine, angiotensin II, phenylephrine, and bradykinin) [58, 59]. 2-APB has been used to block IP3 receptor-induced responses by endothelin, insulin-like growth factor, adenophostin, and IP3 esters themselves in cardiac myocytes [60–64], without affecting IP3 production.

Our data clearly demonstrate for the first time that pre-treatment with gentamicin and/or 2-APB, can significantly reduce the number of TUNEL positive cardiomyocytes and increase the number of viable myocytes following treatment with U46619. These data suggest that IP3 signaling may play a role in TXA2-mediated death of cardiomyocytes, and nicely complement our previous work demonstrating gentamicin and 2-APB prevent TXA2R mediated increases in intracellular Ca^2+^ and ventricular arrhythmogenesis [10, 16]. Collectively, our data suggests that eliminating the deleterious effects of TXA2R signaling along this pathway should significantly improve patient outcomes with respect to ventricular arrhythmias and cardiac remodeling. Moving forward, there is strong rationale to pursue IP3 signaling as an active participant in the death of cardiomyocytes and the remodeling of the heart.

Based on our and other laboratories’ previous findings with TXA2 and the heart as well as these current findings, we propose the following general hypothesis and model of action for TXA2 during pathological increases in the heart (such as that which occurs during myocardial ischemia). Acute TXA2R activation (within seconds to minutes) may increase intracellular calcium which can directly induce arrhythmias as we have demonstrated in vivo [16] and we and others have shown in vitro [16, 52]. While chronic TXA2R activation (hours to days) likely induces cell death over cardiac hypertrophy. We propose that hypertrophy associated with TXA2 observed in the Zhang et al. study [26] may be, at least in part, an adaptive response to overcome a loss of contractility induced by TXA2-mediated cell death. Once cardiomyocytes have died, infiltration of immune cells may remodel the heart and the remainder of the cardiomyocytes likely hypertrophy in an effort to maintain cardiac output. This is supported by a study showing that TXA2R activation triggers immune cell infiltration associated with cardiomyocyte remodeling following cell death [24]. Over time, the net effect of this remodeling may reduce ejection fraction and fractional shortening as typically observed during heart failure and that has been observed in other studies [24, 26]. However, more experiments will need to be conducted to fully understand this process and to continue to test this hypothesis.

Given the indirect (platelet aggregation and vasoconstriction) and direct (disrupted Ca2+ homeostasis, arrhythmogenesis, and cell death) effects TXA2 has on the cardiovascular system, we believe that treatments aimed at directly countering the direct effects of TXA2 may be warranted. Currently, standard therapy recommends the use of aspirin to reduce TXA2 levels. However, it is an over-simplification to conclude that aspirin treatment would eliminate the concern over TXA2’s direct effects on the myocardium. In fact, it is estimated that almost a third of the population is aspirin resistant [65], with TXA2 synthesis and/or TXA2R activation being readily detected despite aspirin therapy [66, 67]. Chronic low-dose aspirin treatment may in some cases be harmful since this has been shown to actually up-regulate TXA2R expression [68], which may make patients more susceptible to the deleterious effects of TXA2. Therefore, more specific interventions (such as IP3) may be needed to prevent TXA2-induced remodeling.

## Conclusions

The findings of this manuscript demonstrate that TXA2 alone does not directly induce changes in cardiac hypertrophy, or genes associated with pathological hypertrophy. However, we have demonstrated that TXA2R signaling is capable of inducing cell death and DNA fragmentation. Importantly, we provide evidence that direct cardiomyocyte TXA2R stimulation of IP3 pathways may play a role in mediating cell death in cardiomyocytes.

## Abbreviations

TXA2: Thromboxane A2
TXB2: Thromboxane B2
TXA2R: Thromoxane A2 receptor
PLC: Phospholipase C
IP3: Inositol trisphosphate
AVCMs: Adult ventricular cardiomyocytes
BDM: 2,3-butanedione monoxime
2APB: 2 aminoethyl diphenylborate
MTT: 3-[4,5-dimethylthiazol-2-yl]-2,5-diphenyltetrazolium bromide
COX2: Cyclooxygenase 2.
